# Anticoagulant therapy and altered tissue factor expression protect against experimental placental and cerebral malaria

**DOI:** 10.1371/journal.ppat.1013259

**Published:** 2025-07-03

**Authors:** Alicer K. Andrew, Tara C. Bracken, Nicole Nazario-Maldonado, Brittany N. Russ, Kathyrn T. Gonzalvo, Benjamin M. Brainard, Nigel Mackman, Julie M. Moore

**Affiliations:** 1 Department of Infectious Diseases, College of Veterinary Medicine, and Center for Tropical and Emerging Global Diseases, University of Georgia, Athens, GeorgiaUnited States of America; 2 Department of Infectious Diseases and Immunology, College of Veterinary Medicine, University of Florida, Gainesville, Florida, United States of America; 3 Department of Small Animal Medicine and Surgery, College of Veterinary Medicine, University of Georgia, Athens, Georgia, United States of America; 4 Department of Medicine, Division of Hematology, UNC Blood Research Center, University of North Carolina at Chapel Hill, Chapel Hill, North Carolina, United States of America; Columbia University Irving Medical Center, UNITED STATES OF AMERICA

## Abstract

Severe malaria remains a major public health concern in regions of moderate to high *Plasmodium falciparum* transmission. Women and young children are especially vulnerable to two clinical manifestations of severe *P. falciparum* malaria, known as placental malaria (PM) and cerebral malaria (CM). Both PM and CM have been characterized as procoagulant states; however, the role of coagulation in galvanizing poor health outcomes is incompletely understood. Moreover, the contribution of tissue factor (TF), the primary driver of the extrinsic pathway of coagulation, to the pathogenesis of PM and CM has not been fully explored. This work utilizes experimental murine models of PM (EPM) and CM (ECM) to explore the impact of anticoagulant treatment and tissue-specific or global reduction in TF expression on disease outcomes. In EPM, we show that treatment of wild-type mice with dalteparin, an anticoagulant class that is safe to use in humans during pregnancy, prevented malaria-induced pregnancy loss, significantly improved embryo viability, and decreased placental fibrin deposition at midgestation. Similarly, mice deficient for endothelial/hematopoietic TF, TF^Tie2Δ^, exhibited a superior ability to maintain their pregnancies at midgestation compared to TF-intact littermate controls, who unequivocally lost their pregnancies. Uterus weight and embryo viability were significantly improved in TF^Tie2Δ^ dams despite experiencing similar parasite burdens as controls. In ECM, dalteparin treatment promoted preservation of the blood-brain barrier (BBB) and protected against the development of neurological signs. Likewise, mice genetically modified to have low TF expression (LTF) exhibited less perivascular leakage in the brain and significantly increased survival probability compared to their littermate controls (TFhet). Together, these data show that anticoagulant treatment can successfully protect against poor health outcomes in two murine models of severe malaria and identify a potentially universal role of TF in driving severe malaria pathogenesis.

## Author summary

Despite decades of research, the mechanistic drivers of severe malaria development are not well understood. Mouse models of severe disease, though imperfect, are an invaluable resource for investigating the underlying causes of severe disease, especially given the insurmountable ethical and logistical challenges of conducting mechanistic studies in human populations. Using two well established mouse models of experimental placental (EPM) and cerebral malaria (ECM), we have found that coagulation and expression of the procoagulant molecule, tissue factor (TF), are central contributors to poor health outcomes associated with these malaria syndromes. Our findings suggest that TF may represent a universal driver of severe disease development, and that anticoagulant therapy that targets some downstream consequences of TF activity, may be useful in protecting against severe outcomes.

## Introduction

Malaria poses a severe threat to global health, with over 249 million cases reported in 2022 in 85 malaria-endemic countries [1]. Pregnant women and young children living in regions of high malaria transmission are the most vulnerable to two clinical manifestations of severe disease, placental malaria (PM) and cerebral malaria (CM), respectively. The syndromes of PM and CM are characterized by the sequestration of *Plasmodium*-infected erythrocytes (IE) in the blood spaces of the placenta and the brain microvasculature, respectively, resulting in dysregulated hemostasis and procoagulant and proinflammatory states [2–7].

PM pathogenesis involves the specific adherence of IE to the syncytiotrophoblast (ST), a specialized fetally-derived epithelial layer that lines the placental intervillous space at the maternal-fetal interface. IE adherence to the ST is facilitated by the expression of a highly polymorphic *Plasmodium falciparum*-derived protein called VAR2CSA [8–11]. VAR2CSA binds to chondroitin sulfate A (CSA) present on the ST, resulting in altered ST function, proinflammatory cytokine and chemokine secretion by the ST, and parasite evasion from immune clearance by the spleen [12–14]. Maternal responses to infection can also contribute to PM pathogenesis through immune cell recruitment, elevated proinflammatory cytokine production, dysregulated hemostasis, fibrin deposition, and lipid peroxidation in the placenta [15–19]. Malaria pigment, known as hemozoin, may also be involved in pathogenesis, as it is commonly found in the placenta during PM and is capable of stimulating cytokine production by the ST and promoting immune cell dysfunction *in vitro* [13,20–22].

Clinical consequences of PM include maternal anemia, fetal growth restriction, preterm delivery (before 37 weeks gestation), and low birth weight (LBW, < 2500g), with prematurity and LBW being strong risk factors for perinatal, neonatal, and infant morbidity and mortality [23–26]. In 2022, roughly 12 million pregnancies were exposed to *P. falciparum* infection in Sub-Saharan Africa, resulting in approximately 393,000 LBW deliveries [1,27–29]. The World Health Organization (WHO) estimates that the use of intermittent preventative treatment in pregnancy (IPTp) averted LBW in about 512,000 neonates in 2022; however, achieving high coverage of the WHO-recommended three doses of IPTp (IPTp3) remains challenging, limiting the impact of IPTp on LBW in some settings [30]. With IPTp3 coverage continuing to fall well below global targets and emerging parasite resistance against frontline antimalarials, PM remains a substantial health burden in endemic regions [1,27]. These challenges emphasize the importance of identifying host-directed therapies that may mitigate PM-associated poor health outcomes.

Like PM, CM poses a significant health threat in vulnerable populations. CM is defined by the presence of *P. falciparum* parasitemia and unrousable coma (Blantyre scale ≤2), with no other apparent etiology [31]. Although the precise pathogenic mechanism(s) of CM are unclear, sequestration and cytoadherence of IE to endothelial cell proteins in the brain microvasculature have been commonly reported in postmortem studies of CM [2,32]. One such endothelial protein, endothelial protein C receptor (EPCR), acts as a receptor for variants of the *P. falciparum* sequestration ligand PfEMP1 that are associated with severe disease, an interaction that disrupts EPCR anticoagulant and endothelial cytoprotective activity [33,34]. IE sequestration is further associated with endothelial cell activation and dysfunction, increased blood-brain barrier (BBB) permeability, and localized inflammatory responses [35–37]. Perivascular hemorrhage, fibrin deposition, and BBB breakdown have also been observed near sites of IE cytoadherence [36,38,39].

Clinically, CM is a hypercoagulable condition, with the degree of coagulation activation correlating with disease severity [40,41]. Current treatments for CM rely on the use of antimalarial drugs and supportive care [42–44]. Even with early treatment, CM still carries a case fatality rate between 15–20%, and those who recover from the illness may still experience long-term neurological impairments [43,45,46]. Therefore, studies aimed at elucidating the pathogenesis of severe and fatal CM are needed to improve future therapeutic strategies.

Given the challenges of studying PM and CM in endemic populations, murine models of malaria infection that share common features with these human syndromes are important tools for advancing our understanding of severe malaria pathogenesis. Indeed, a procoagulant state and dysregulated hemostasis have been reported in both human and murine PM and CM studies [6,19]. For instance, disseminated intravascular coagulation (DIC) has been associated with fatal outcomes in pediatric CM and decreased expression of the anticoagulant endothelial protein C receptor may exacerbate the procoagulant state in CM in both humans and mice [47–50]. In PM, fibrin deposition is a hallmark of PM+ placentae in humans and mice [19,51–53] and histologic fibrin scoring is negatively associated with birth weight [19]. Interestingly, in an experimental model of PM using *Plasmodium chabaudi-*infected pregnant mice, anticoagulant treatment with research-grade low molecular weight heparin (LMWH) and enoxaparin (FDA-approved LMWH) reduced placental pathology and fibrin deposition and enabled dams to maintain pregnancy through midgestation [19]. In the same model system, treatment with a neutralizing antibody against tumor necrosis factor alpha (TNF) resulted in a similarly protective effect, with the noteworthy observation that treated mice had reduced placental expression of the procoagulant protein, tissue factor (TF) [54].

TF is a transmembrane protein and the primary initiator of coagulation [55]. It is expressed by several cell types and may function as a molecular connection between malaria-induced coagulation, endothelial activation, and possibly proinflammatory cytokine production [56]. IE induce endothelial cell activation and TF expression *in vitro*, which may contribute to hypercoagulability during severe malaria [56,57]. In PM, macrophages expressing TF were associated with fibrin deposition in infected placentae and TF-bearing monocytes were observed in the intervillous blood space [58]. In CM, TF immunolocalized to endothelial cells is associated with the presence of thrombi and hemorrhage in the brain [57]. However, a more complete understanding of the participation of TF in severe malaria pathogenesis is needed to elucidate the mechanisms that galvanize poor health outcomes in PM and CM.

Despite associations between a procoagulant state and disease severity in PM and CM, no previous study has formally described a potentially universal mechanism for disease pathogenesis *in vivo*. Our study sought to investigate the extent to which TF expression represents a shared driver for experimental PM (EPM) and CM (ECM) pathogenesis. We hypothesized that therapeutic inhibition of coagulation and a direct reduction in TF expression could effectively modulate procoagulant-induced adverse health outcomes associated with EPM and ECM. The findings from our study position TF and its downstream effects on coagulation as important mediators of severe malaria pathogenesis.

## Results

### Anticoagulant therapy significantly improves pregnancy outcomes in EPM

**Fig 1:**
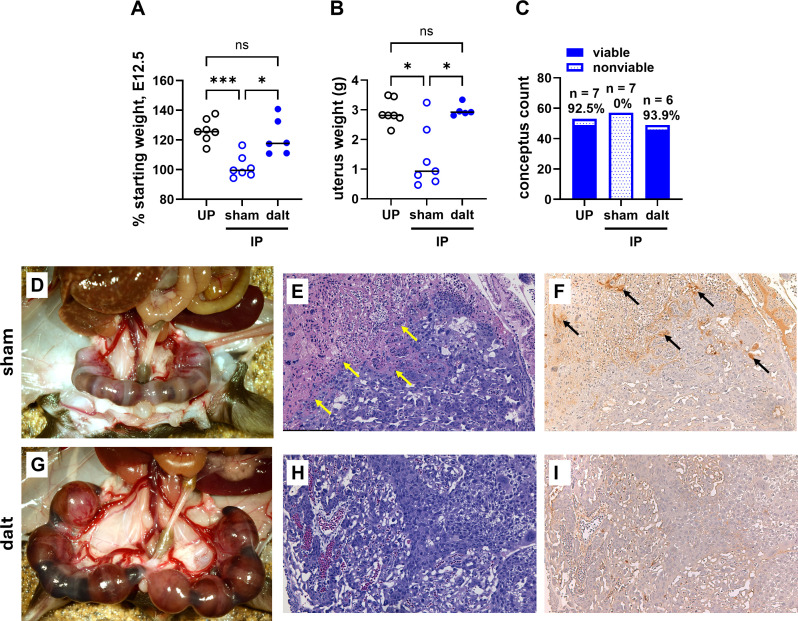
Anticoagulant treatment improves pregnancy outcomes at E12.5 in EPM. *P. chabaudi chabaudi* AS-infected B6 dams were treated daily with dalteparin (dalt) or received sham-treatment from E6.5 to E12.5. (A) Percent starting weight, (B) uterus weight, and (C) conceptus count of sham-treated (sham, n = 7) and dalteparin-treated (dalt, n = 6) infected pregnant (IP) B6 dams and uninfected pregnant (UP) controls (n = 7) at E12.5. (D) Gross pathological images of the uterus in sham-treated IP and (G) dalteparin-treated IP mice displaying marked improvement in embryo viability in the dalteparin-treated IP group. (E) H&E and (H) fibrin(ogen)-stained serial sections from sham-treated IP placenta show pronounced tissue disruption and fibrin(ogen) compared to (H, I) dalteparin-treated IP placenta. Yellow arrows indicate areas of necrosis in the placenta. Black arrows indicate areas of intense fibrin(ogen) staining in blood spaces. Bars represent the median, ***P = 0.0002, *P < 0.03, ns = not significant, ANOVA with Dunnett’s T3 multiple comparisons test (A, B), chi-square test (C).

Guided by previous studies using LMWH treatment to mitigate *Pcc*AS-induced midgestational pregnancy loss [19], infected pregnant (IP) B6 mice were treated daily from E6.5 to E10.5 with 1000 IU/kg of an FDA-approved LMWH, dalteparin. On E12.5, sham and dalteparin-treated IP mice were euthanized, and pregnancy outcomes were assessed. At euthanasia, dalteparin-treated IP mice exhibited significantly higher weight as a proportion of starting weight compared to their sham-treated IP counterparts (Fig 1A). Similarly, uterus weights at E12.5 in dalteparin-treated IP mice were significantly higher compared to the sham-treated IP controls (Fig 1B). Embryos in sham-treated IP mice were universally nonviable whereas embryo viability in dalteparin-treated IP dams was equivalent to UP controls (Fig 1C). Furthermore, E12.5 embryos were macerated and necrotic in the sham-treated IP dams (Fig 1D), while embryos in the dalteparin-treated IP dams remained largely intact (Fig 1G). Interestingly, while parasitemia did not differ between the sham-treated and dalteparin-treated IP dams, hematocrit at E12.5 was higher in the latter, despite a faster decline during ascending parasitemia (S1 Fig). The protective effect of dalteparin was further confirmed by histological analysis of placentae. Sham-treated IP placentae were characterized by large areas of necrosis and degradation of placental architecture that were not observed in the dalteparin-treated IP mice (Fig 1E and 1H). Additionally, immunohistochemical staining demonstrated increased fibrin(ogen) deposition in sham-treated IP relative to dalteparin-treated IP placenta (Fig 1F and 1I). These data suggest that therapeutic modulation of the maternal coagulation response with dalteparin is sufficient to prevent adverse pregnancy outcomes at midgestation in the *Pcc*AS model of EPM.

### Anticoagulant therapy significantly improves blood-brain barrier integrity in ECM

**Fig 2:**
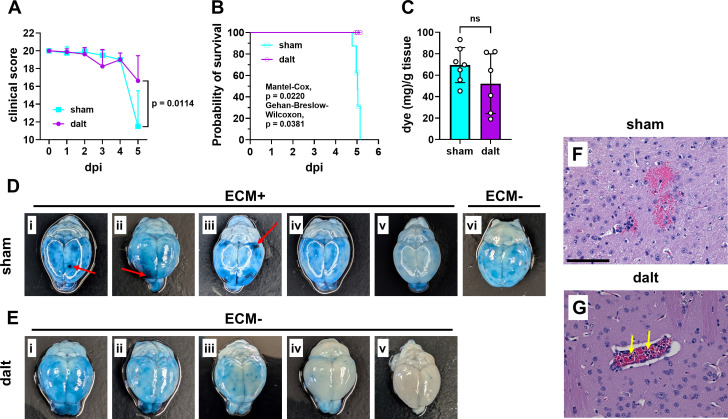
Anticoagulant treatment enhances blood-brain barrier integrity in ECM. *P. berghei* ANKA-infected B6 mice were treated daily with dalteparin (dalt) or received sham-treatment from the day of infection until 5dpi. (A) Results from a 10-parameter clinical assessment rubric show a significant decline in clinical scores for sham-treated relative to dalteparin-treated infected mice. (B) Probability of survival analysis depicts that 6/8 sham-treated mice developed neurological signs (the other 2 were censored at the end of the experiment with no signs of ECM) and 0/8 dalteparin-treated mice developed neurological signs. (C) Comparison of extracted Evans blue dye quantitation from a sagittal section of cerebrum and cerebellum, inclusive of an olfactory bulb, shows no statistically significant difference between sham-treated and dalteparin-treated mice. (D) Brains from sham-treated mice at 5 dpi display consistent evidence of dye leakage, with several cases of severe vascular leakage, indicated by red arrows. (E) Brains from dalteparin-treated mice show limited signs of severe vascular leakage and, in some cases, little to none. (F) Perivascular bleeding was detected by H&E staining in the brains of sham-treated mice, while (G) blood vessel integrity was maintained in dalteparin-treated mice, with yellow arrows indicating the presence of immune cells in the intact brain vasculature. (A) Two-way repeated measures ANOVA of all time points and Welch’s t-test of 5 dpi data only, (B) symbols represent mice that were censored and euthanized at the time points indicated in the text, (C) Welch’s t-test, ns = not significant, (F) scale bar = 100µm.

To evaluate the efficacy of dalteparin treatment on disease progression in another model for severe malaria, we turned to a widely employed ECM model. In this system, *Pb*A causes a disease that recapitulates important characteristics of human CM, including neurological impairments, BBB disruption, high expression of inflammatory mediators, and coma [3,32,59]. As employed here, this model resulted in development of neurological signs of disease in B6 mice beginning at 5 days post-infection (dpi). First, B6 mice were treated intraperitoneally once daily with 1000IU/kg dalteparin from 2 to 4 dpi. By 5 dpi, all sham-treated mice (4/4, 100%) exhibited neurologic impairment, while only 2 out of 5 dalteparin-treated mice succumbed to ECM. In a larger trial, B6 mice were treated intraperitoneally once daily with 1000IU/kg dalteparin from the day of infection to 5 dpi, before the trial was terminated. Clinical scores, based on a ten-parameter rubric (S1 Table), were equivalent in the dalteparin-treated and sham-treated groups until 5 dpi, when a significant decline, indicative of increased morbidity, was evident in the sham-treated mice (Fig 2A). Consistent with this observation, 6 of 8 sham-treated mice developed neurological signs consistent with ECM, while, in contrast, none of the dalteparin-treated mice (0/8) exhibited ECM at 5 dpi (Fig 2B). Evans blue dye extractions from a sagittal section of brain tissue, used as a measure of BBB permeability, did not reveal a statistically significant difference between the groups (Fig 2C). However, dye staining was evident in all sham-treated mice, regardless of ECM outcome (Fig 2Di-vi). Large areas of intense dye leakage into the brain parenchyma (Fig 2Di-iii, red arrows) and olfactory bulbs (Fig 2Di-v) were observed. In contrast, dalteparin-treated mice (Fig 2Ei-v), none of whom developed ECM, did not show large areas of cerebral dye leakage, and, in two cases (Fig 2iv and 2v), limited dye leakage was observed. Consistent with these observations, perivascular leakage in sham-treated mice was noted in H&E-stained brain sections (Fig 2F) while limited perivascular leakage was evident in the cerebral parenchyma of dalteparin-treated mice, despite immune cell accumulation (Fig 2G). These experiments were performed in an aggressive model of ECM; thus, these findings highlight the remarkable ability of dalteparin to effectively prevent neurological disease development and promote BBB preservation during *Pb*A infection.

### Maternal tissue factor expression contributes to pregnancy loss in EPM

**Fig 3:**
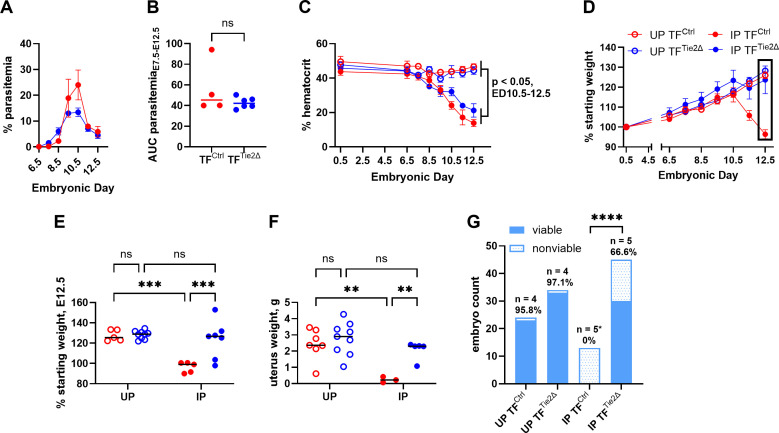
Tie2cre-driven tissue factor deletion mitigates pregnancy loss at E12.5 in EPM. TF-intact (TF^Ctrl^) and endothelial/hematopoietic TF-deficient (TF^Tie2∆^) dams were infected with *Pcc*AS on E0.5 and monitored until the experiment was terminated at E12.5. (A) Percent parasitemia in IP TF^Ctrl^ and IP TF^Tie2∆^ dams, n = 4 and n = 6, respectively. (B) Area under the curve (AUC) of parasitemia from E7.5 to E12.5 in IP TF^Ctrl^ (n = 4) and IP TF^Tie2∆^ (n = 6) dams. (C) Hematocrit in IP TF^Ctrl^ and IP TF^Tie2∆^ dams (D) Percent starting weight in UP TF^Ctrl^, n = 8; UP TF^Tie2∆^, n = 9; IP TF^Ctrl^, n = 6; IP TF^Tie2∆^, n = 7 dams. (E) Percent of starting weight at E12.5 in UP TF^Ctrl^, n = 5; UP TF^Tie2∆^, n = 9; IP TF^Ctrl^, n = 6; IP TF^Tie2∆^, n = 7 dams. (F) Uterus weight of UP TF^Ctrl^, n = 7; UP TF^Tie2∆^, n = 9; IP TF^Ctrl^, n = 3; IP TF^Tie2∆^, n = 5 dams at E12.5. (G) Conceptus counts in IP TF^Ctrl^ and IP TF^Tie2∆^ dams at E12.5; *indicates that conceptuses could be counted in only two dams, due to widespread hemorrhaging and necrosis in the uteri of most IP TF^Ctrl^ dams at this time point. ****P < 0.0001, ***P < 0.001, **P < 0.01, ns = not significant, (B) unpaired t-test with Welch’s correction, (C) mixed effects ANOVA, (E) two-way ANOVA with Fisher’s LSD test, (F) Fisher’s exact test.

Given the success of anticoagulant therapy in protecting IP mice from pregnancy loss at midgestation, further studies were performed to directly interrogate the role of coagulation in this model, with a specific focus on TF. Mice genetically modified to lack expression of TF from endothelial and hematopoietic cells (TF^Tie2∆^) were compared to TF-intact controls (TF^Ctrl^) following infection with *Pcc*AS on E0.5. The course of infection was equivalent in the two strains, indicating that parasitemia was not influenced by genotype (Fig 3A and 3B). Likewise, though significantly decreased in IP mice relative to UP controls, hematocrit, as a proxy for anemia, was similar between the two IP groups (Fig 3C). In UP TF^Tie2∆^ and UP TF^Ctrl^ mice, steady weight gain was observed throughout the experiment, as expected during normal pregnancy (Fig 3D). IP TF^Tie2∆^mice maintained their weight at levels comparable to both UP groups, while, in contrast, IP TF^Ctrl^ mice exhibited precipitous weight loss after E10.5 (Fig 3D). At E12.5, IP TF^Tie2∆^ dams weighed significantly more than IP TF^Ctrl^ dams and were not significantly different from the controls (Fig 3E). Correspondingly, IP TF^Tie2∆^ dams maintained healthier pregnancies overall compared to their IP TF^Ctrl^ counterparts, as evidenced by significantly heavier uteri (Fig 3F) and a greater proportion of viable embryos at E12.5 (Fig 3G). No IP TF^Ctrl^ dams had viable embryos at E12.5 (Fig 3G) and total embryo count at E12.5 was significantly lower in IP TF^Ctrl^ relative to UP TF^Ctrl^ and IP TF^Tie2∆^ dams (S2 Fig). Though IP TF^Tie2∆^ dams had mixed outcomes in embryo viability (66% overall; Fig 3G), with 3/7 having poor viability and 4/7 having 85–100% viability (S2 Table), embryo count was equivalent to UP TF^Tie2∆^ dams (S2 Fig). Notably, if TF expression by the embryo and placenta were a critical determinant of embryo survival in this model, then 50% embryo viability would be predicted for IP TF^Ctrl^ dams and 75% viability in IP TF^Tie2∆^ dams (S3 and S4 Figs). Thus, the results suggest that the dam genotype, ergo TF expression, as opposed to pup genotype, is a critical determinant of pregnancy outcome in this model.

**Fig 4:**
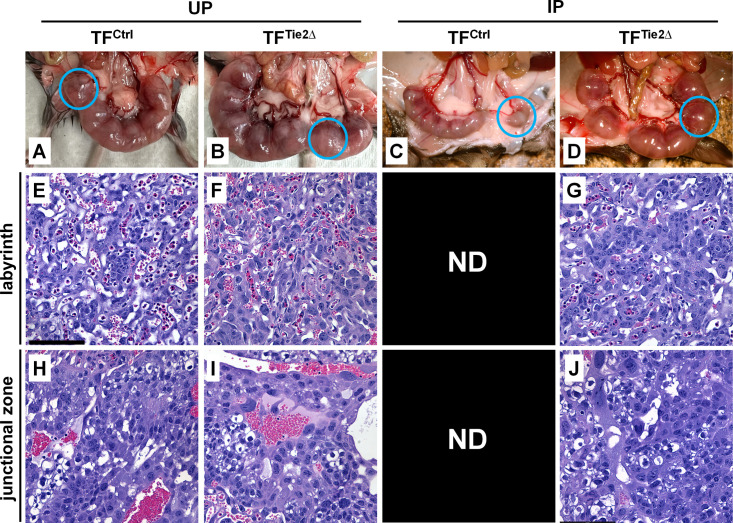
Tie2cre-driven tissue factor deletion preserves midgestational embryo viability and placental integrity in EPM. Pregnancy outcomes were assessed at E12.5 in TF-modified dams infected at E0.5 with *Pcc*AS. (A) Gross pathological images of the uterus in UP TF^Ctrl^, (B) UP TF^Tie2∆^, (C) IP TF^Ctrl^, and (D) IP TF^Tie2∆^ dams at E12.5. Blue circles drawn around individual conceptuses demonstrate the relatively small size and altered morphology of conceptuses in IP TF^Ctrl^ dams compared to the other groups. (E-G) Labyrinth and (H-J) junctional zones in the placenta of UP TF^Ctrl^, UP TF^Tie2∆^, and IP TF^Tie2∆^ dams. ND, not done, indicating that placenta could not be examined due to pregnancy loss in IP TF^Ctrl^ dams at E12.5. Scale bar, 122.5µm.

Gross pathological examination further confirmed that pregnancy outcome in IP TF^Ctrl^ dams was poor relative to IP TF^Tie2∆^ dams (Fig 4). IP TF^Tie2∆^ embryos (Fig 4D) were healthy and comparable in size to UP embryos (Fig 4A and 4B), whereas IP TF^Ctrl^ embryos were growth-restricted, macerated, and nonviable (Fig 4C). Profound tissue degradation of the embryos in the latter group precluded histological evaluation at E12.5. Examination across the remaining groups revealed that placental architecture in both the labyrinth and junctional zone was maintained in IP TF^Tie2∆^ mice (Fig 4G, J) and was comparable to UP controls at the same time point (Fig 4E, 4F, 4H, and 4I). Together, these data suggest that TF expression in endothelial cells/hematopoietic cells plays a key role in embryo outcome in *Pcc*AS infection, but at the level of the dam, and not in the embryo and/or placenta.

### Low tissue factor expression protects against ECM and improves blood-brain barrier integrity

**Fig 5:**
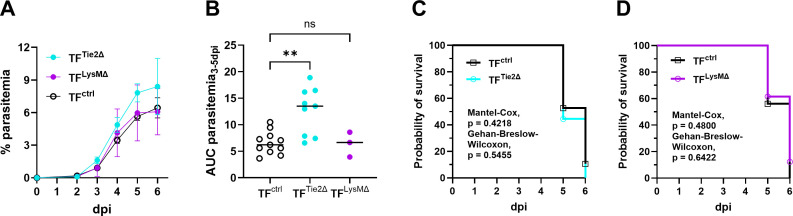
Tissue factor deletion on hematopoietic/endothelial and myeloid cells did not improve disease outcomes in ECM. Parasitemia and probability of survival were assessed in *Pb*A-infected TF^Tie2∆^, TF^LysMΔ^, and TF^Ctrl^ mice. (A) Percent parasitemia in *Pb*A-infected Tie2cre-driven tissue factor deficient (TF^Tie2∆^), LysM-driven tissue factor deficient (TF^LysMΔ^), and tissue factor intact (TF^Ctrl^) mice; TF^Tie2∆^, n = 22; TF^LysMΔ^, n = 11; TF^Ctrl^, n = 13 (TF^Tie2∆^ littermates), n = 18 (TF^LysMΔ^ littermates). (B) Area under the curve (AUC) of parasitemia 3–5 dpi in TF^Ctrl^, TF^Tie2∆^, and TF^LysMΔ^ mice. (C) Probability of being ECM-free in TF^Ctrl^ vs TF^Tie2∆^ and (D) TF^Ctrl^ vs TF^LysMΔ^ mice. (B) **P = 0.0059, one-way ANOVA with Dunnett’s T3 multiple comparisons test, ns = not significant. (C, D) Statistical tests are as shown in the panels.

Next, we assessed the impact of TF expression on susceptibility to ECM, by initiating *Pb*A infection in mice with cell type-specific deletion of TF. First, we evaluated the development of ECM in TF^Tie2∆^ and TF^LysM∆^ mice that are deficient for TF on endothelial/hematopoietic and myeloid cells, respectively. Though TF^Tie2∆^ mice developed parasitemia more rapidly than TF^Ctrl^ and TF^LysM∆^ mice (Fig 5A), resulting in a significantly higher peripheral parasite burden over the course of the experiment (Fig 5B), susceptibility to ECM in TF^Tie2∆^ and TF^LysM∆^ mice was indistinguishable from TF intact (TF^Ctrl^) controls (Fig 5C and 5D).

**Fig 6:**
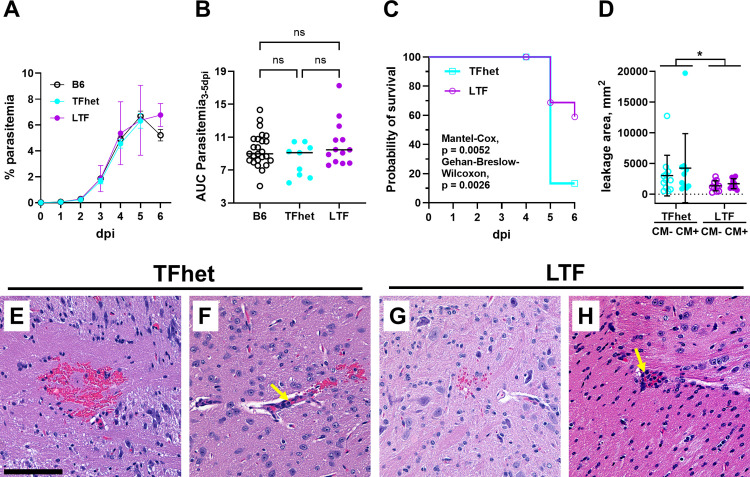
Low global tissue factor expression is associated with increased probability of survival during ECM and limits brain perivascular leakage. Parasitemia and probability of survival were assessed in *Pb*A-infected wildtype B6, LTFhet, and LTF mice. (A) Percent parasitemia in *Pb*A-infected B6, low tissue factor heterozygotic (mTF^+/-^, hTF^+^ or TFhet), and low tissue factor null (mTF^-/-^, hTF^+^ or LTF) mice; B6, n = 60; TFhet, n = 20; LTF, n = 16. (B) Area under the curve (AUC) of parasitemia 3–5 dpi in B6, TFhet, and LTF mice. (C) Probability of remaining ECM-free in TFhet (n = 19) and LTF (n = 16) mice. (D) Histological measurement of perivascular leakage in ECM- (CM-) and ECM+ (CM+) TFhet and LTF brains. Ten high power fields with at least one area of leakage were analyzed for one mouse brain of each outcome and genotype. (E, F) H&E-stained sections of ECM + TFhet and (G, H) ECM + LTF cerebral cortex at 5 dpi. (F, H) Yellow arrows point to inflammatory cells visible in the brain microvasculature of both strains. (B) Welch’s ANOVA with Dunnett’s T3 post hoc multiple comparisons test, ns = not significant. (D) Two-way ANOVA, *P = 0.0422. Bar, 100 μm.

ECM outcome was next assessed in mice with a global reduction in TF expression (Low Tissue Factor, “LTF”). In these experiments, the controls were “TFhet”, which express 50% levels of TF. Neither course of infection (Fig 6A) nor parasite burden (Fig 6B) up to 5 dpi differed among B6, TFhet, and LTF mice. The majority of TFhet mice succumbed to ECM by 5 dpi whereas over half of LTF mice survived to 6 dpi without developing any neurological signs (Fig 6C). H&E-stained thin sections of LTF and TFhet brain were examined for the presence of histopathologic features characteristic of ECM. Perivascular leakage was elevated in TFhet brain relative to LTF brain, regardless of development of ECM (Fig 6D, P = 0.0422 by two-way ANOVA). Areas of leakage in the TFhet brain with ECM extended well beyond the adjacent vasculature (Fig 6E), while leakage area was relatively reduced in the LTF brain with ECM (Fig 6G). In both, inflammatory cells in in the vascular lumen were evident (Fig 6F and 6H). All together, these results suggest that while TF on endothelial and some hematopoietic cells (TF^Tie2∆^) and myeloid cells (TF^LysM∆^) does not contribute to ECM pathogenesis in this model, TF provided by an alternate cell type is a critical driver of disease.

## Discussion

In these studies, we used anticoagulant treatment and genetic manipulation to identify a universal role for coagulation, and more specifically TF expression, in two experimental models of severe malaria. In the EPM model, we found that dalteparin treatment enabled *Pcc*AS-infected pregnant B6 mice to avoid malaria-induced pregnancy loss at midgestation. Dalteparin-treated dams had significantly heavier uterus weights compared to sham-treated controls, which corresponded to a significant increase in embryo viability at midgestation. Histopathologic analysis revealed significant areas of necrosis and fibrin deposition in the placentae of sham mice, pathologies that were not present in the anticoagulant-treated mice. These data corroborate our previous report indicating that anticoagulant therapy can effectively improve pregnancy outcomes in EPM [19].

Dalteparin also exerted a protective effect in ECM. Treatment significantly mitigated BBB permeability in mice at 5 dpi, as evidenced by the overall reduction in Evans blue dye leakage into the brain parenchyma in treated mice relative to sham-treated controls. Histopathology confirmed this protective effect by revealing signs of hemorrhage in sham-treated mice that were not observed in the treated counterparts, despite evidence of accumulated inflammatory cells in the brain microvasculature. In addition to BBB preservation, none of the anticoagulant-treated mice developed ECM while the majority of the sham-treated mice succumbed to disease. Treatment success in this aggressive model featuring rapid onset and ECM development by 5 or 6 dpi is encouraging. Considered together with other studies, including an alternate model of ECM using *Pcc*AS*-*infected IL-10^-/-^ mice in which significantly improved survival was observed following treatment with the LMWH enoxaparin sodium [60], compel future studies to further evaluate the potential therapeutic benefit of anticoagulant therapy during severe malaria.

An obvious mechanistic basis for anticoagulant treatment-driven improvements in EPM and ECM is inhibition of the coagulation cascade. Indeed, enoxaparin treatment in the *Pcc*AS/IL-10^-/-^ mouse model for ECM reduced fibrin(ogen) deposition in the brain vasculature nearly to uninfected control levels [60]. We and others [19,56,57] have hypothesized that pathologic thrombosis in severe malaria is driven by the initiator of the extrinsic coagulation cascade, TF. Here, we demonstrate that maternal TF plays a significant role in EPM pathogenesis and EPM-driven pregnancy loss. Mice with TF depleted from endothelial/hematopoietic cells (TF^Tie2∆^) maintained pregnancy during malaria infection whereas their TF-intact (TF^Ctrl^) controls experienced significant compromise of pregnancy. Remarkably, IP TF^Tie2∆^ mice avoided pregnancy loss at peak parasitemia, despite having similar parasite burdens as their IP TF^Ctrl^ littermates. TF deficiency also significantly improved embryo viability at midgestation, resulting in uterus weights that were indistinguishable from UP dams. Gross pathologic assessment confirmed that IP TF^Tie2∆^ dams had healthier embryos after peak parasitemia at E12.5, without obvious signs of placental disruption or pathology. These data suggest that maternal endothelial/hematopoietic TF is an important mediator of malaria-induced pregnancy compromise in EPM. Since the Tie2/Tek promoter is active in both endothelial and hematopoietic cells [61,62] and in the placental trophoblast [63,64], TF deletion in any one or combination of these cell types may drive the pregnancy rescue phenotype described here. However, it is important to note that the experimental crosses utilized in these experiments are predicted to yield mixed embryo genotypes (either 50% TF intact and 50% TF depleted in TF^Ctrl^ dams or 25% TF intact and 75% TF depleted in TF^Tie2∆^ dams; see S3 and S4 Figs), yet embryo genotypes seemed to have no impact on pregnancy outcomes, given that all embryos from TF^Ctrl^ dams were nonviable and most embryos from TF^Tie2∆^ dams were viable. Thus, embryo viability was linked to the dam genotype, while TF expression by the conceptus, or lack thereof, does not appear to be an essential factor for the phenotypes observed in this work. Nonetheless, a lack of an apparent role for trophoblast-derived TF here is at odds with our previous work in this EPM model that found significantly reduced trophoblast TF expression in placentae of mice treated with anti-TNF antibodies, which rescued midgestational pregnancy [54]. Ultimately, more studies are needed to further explore the cell types and anatomical sites in which TF expression contributes to pathogenesis in EPM.

In contrast to the EPM findings, neither myeloid nor endothelial/hematopoietic TF deletion had an effect on ECM onset or survival, prompting our investigation of the role for global TF expression in ECM development. We found that LTF mice had a significantly higher probability of survival compared to their TFhet and B6 counterparts, which was associated with less perivascular leakage in the brain, despite similar parasite burdens across groups. Interestingly, perivascular leakage was elevated in TFhet mice regardless of ECM outcome and was also similar in ECM- and ECM + LTF mice, suggesting that other factors, in addition to the vascular leakage observed here, are critical to development of neurological signs in these mice.

Although our results together suggest that global TF expression is an important contributor to the development of ECM, future investigations are required to elucidate the relationships and relative importance of inflammation and coagulation in this model and establish the specific TF-expressing cell types involved in ECM outcomes. Of note, astrocytes are known to be the major source of TF in the brain [65,66]. Activation of these cells, and their association with blood vessels containing fibrin deposits, have been reported in the IL-10^-/-^/*Pcc*AS model for ECM [60]. Interestingly, microgliosis is also characteristic of ECM in this model, with a contrasting anticoagulant and protective role [67]. The relative importance of these cells and inflammatory factors and other reported aspects of CM pathogenesis, including IE adherence, endothelial activation, loss of endothelial protein C receptor, and CD8 + T cell migration and accumulation in the brain [2,32,35,49,60,68–70], should be addressed to better characterize the multifactorial nature of CM development [32].

Although experimental models of PM and CM do not perfectly recapitulate severe malaria pathogenesis in all human clinical contexts, our studies provide compelling evidence that coagulation, and in particular, expression of TF, are critical mediators of pathogenesis in EPM and ECM. Future studies should focus on a comprehensive analysis of the effects of anticoagulant treatment on other aspects of severe disease, including IE cytoadherence, dysregulated hemostasis, oxidative stress, and immune cell function in both systems. Studies should also delve deeper into the mechanistic basis of TF-driven EPM and ECM development, including the key cell types that contribute TF activity. The complexity of host responses to PM and CM poses a significant challenge to developing effective therapies against these diseases, and large gaps in knowledge will need to be filled before a complete understanding of the pathological mechanisms underlying these diseases can be formed. However, the experiments presented here provide valuable insight into the procoagulant response to placental and cerebral malaria and demonstrate that TF plays an important role in the pathogenesis of both syndromes.

## Materials and methods

### Ethics statement

All mouse experimentation was conducted following the guidelines and regulations set forth by, and with approvals from, the University of Georgia Animal Care and Use Committee and the University of Florida Institutional Animal Care and Use Committee.

### Mice

C57BL6/J (B6) and A/J mice were originally purchased from The Jackson Laboratory and were used to generate breeding stock and experimental animals in the University of Georgia Coverdell Vivarium. Mice with floxed TF [71] (TF^Ctrl^) were crossed with mice expressing Cre recombinase in different cell types to generate mice with tissue-specific deletions of TF. Mice expressing cre recombinase controlled by the Tie2/Tek promoter have deletion of the TF gene in vascular endothelial cells, hematopoietic cells [61,62], and fetally-derived trophoblast [63] and are referred to as TF^Tie2∆^. TF^Ctrl^ mice lacking cre recombinase are referred to as “controls” and are phenotypically normal [62]. TF^Tie2∆^ mice were generated by mating hemizygous TF^Tie2∆^ studs sourced from the Jackson Laboratory with TF^Ctrl^ dams generously provided by Dr. Nigel Mackman. TF^LysM∆^mice express cre recombinase under the control of the Lyz2 (lysozyme M) promoter and have a specific deletion of the TF gene in myeloid cells [62,72]. Founder TF^LysM∆^ and TF^Ctrl^ breeder pairs were provided by Dr. Mackman and maintained by mating hemizygous TF^LysM∆^ studs with TF^Ctrl^ dams. TF^Tie2∆^ and TF^LysM∆^ mice are viable, fertile, normal in size, and do not display gross physical or behavioral abnormalities compared to wildtype B6 mice. Dr. Mackman also provided “low TF” (LTF) mice that are homozygous null for mouse *F3* and are transgenic for human TF (*F3*^−/−^, hTF+), resulting in TF levels that are ~ 1% of normal levels [73,74]. Heterozygous littermates (*F3*^+/−^, hTF + ; “TFhet”), generated by breeding TFhet females with LTF males, have 50% of normal TF levels with no evidence of hemostatic defects and were used as littermate controls [73]. All lines were maintained under SPF conditions and were *Helicobacter* negative.

All experimental mice used in EPM experiments were virgin females between the ages of 8–10 weeks old. Stud males derived from the same breeders as the experimental virgin females were used until approximately 6 months of age, then replaced by younger males of the same lineage. TF^Tie2∆^ and TF^Ctrl^ control females were bred with TF^Tie2∆^ males, producing an expected ratio of 75% TF^Tie2∆^ and 25% TF^Ctrl^ embryos (S3 Fig) or 50% TF^Tie2∆^ and 50% TF^Ctrl^ embryos (S4 Fig), respectively. All mice used in ECM experiments were 6–7 weeks old virgin females. Experiments were performed in accordance with the guidelines and with the approval of the University of Georgia Institutional Animal Care and Use Committee (Animal Use Protocols numbers A2014 04–002-Y3-A4 and A2015-03–005-Y1-A1).

### Parasites

*Plasmodium chabaudi chabaudi,* Strain AS (*Pcc*AS), MR4–741 (provided by David Walliker) was obtained through the BEI Resource Repository, NIAID, NIH, and maintained as frozen stock in accordance with supplier guidelines. Parasites were routinely passaged in female A/J mice for the purpose of infecting experimental mice [75]. *Plasmodium berghei* ANKA (*Pb*A) parasites constitutively expressing green fluorescent protein (GFP) (*Plasmodium berghei* ANKA GFPcon 259cl2, MRA-865, provided by Chris J. Janse and Andrew P. Waters) were obtained through BEI Resources Repository, NIAID, NIH and maintained by passaging in naïve male C57BL6/J mice. *Pb*A-GFP parasites enabled flow cytometric monitoring of parasitemia in ECM experiments.

### Experimental placental malaria (EPM)

Timed pregnancy experiments and monitoring of experimental mice were performed using a previously established protocol [75]. The day a vaginal plug was observed in time-mated 8- to 10-week-old female mice was considered embryonic day 0.5 (E0.5). On E0.5, mice were infected via tail vein injection with 1000 *Pcc*AS IE diluted in 200ul 1X phosphate-buffered saline (PBS) per 20g of body weight and termed infected pregnant (IP). In the control group, mice were sham injected with 200ul 1X PBS per 20 grams body weight on E0.5 and designated as uninfected pregnant (UP). From E6.5 through E12.5 (which correspond to 6–12 days post-infection (dpi), respectively), each mouse was weighed, and blood was collected from the tail vein into a heparinized capillary tube to measure hematocrit and parasitemia. Flow cytometric analysis was used to monitor parasitemia, following a previously established protocol [76]. In brief, 2μl of infected whole blood was diluted in 98μl 1X PBS and stained with 0.25μl SYTO-16 Green Fluorescent Nucleic Acid Stain (Thermofisher Scientific, catalog # S7578), then analyzed using a Beckman Coulter CyAn ADP Analyzer (Beckman Coulter, Indianapolis, IN, USA). Erythrocytes were gated based on size and fluorescence intensity and parasitemia was defined as the percentage of green fluorescent-positive events occurring in the erythrocyte gate. 100,000 events in the erythrocyte gate were acquired per sample and an uninfected blood sample was used as a negative control. The remaining blood was centrifuged for 3 minutes, and the percent hematocrit was calculated according to the following: (volume of packed erythrocytes)/(total blood volume) x100%.

### Experimental cerebral malaria (ECM)

Infection was initiated in female mice aged 6–7 weeks old via tail vein injection with 1x10^6^ *Pb*A-GFP IE. Mice were monitored up to 6 dpi. Each mouse was weighed daily and blood was collected from the tail vein to measure parasitemia and hematocrit, as described above. A scoring rubric to assess physical, behavioral and neurological parameters was applied daily. From late 4 dpi through 6 dpi, mice were monitored at 3-hour intervals and assessed for neurologic signs consistent with ECM, including tremors, ataxia, impaired righting reflex, limb paralysis, and seizures. Observation of any of these features prompted diagnosis of ECM (ECM+) and humane euthanasia. Mice that did not develop neurological signs are denoted as ECM-negative (ECM-).

### Drug treatment – EPM and ECM

Mice were treated with 1000 IU/kg dalteparin sodium (Fragmin, Pfizer Inc.) or sham-injected with 150μL PBS intraperitoneally every 24 hours. This dosage frequency and level with research-grade LMWH and enoxaparin was found to protect against malaria-induced midgestational pregnancy loss [19].

### Euthanasia and sample collection – EPM

Mice were anesthetized on E12.5 using 2.5% 2,2,2-Tribromoethanol (Avertin, Sigma-Aldrich, St. Louis, MO, USA) administered via intraperitoneal injection or by isoflurane inhalation and euthanized by exsanguination. Alternatively, mice were anesthetized via isoflurane inhalation (Parkland Scientific Rodent Table Top Anesthesia Machine, Coral Springs, FL). Embryo viability was assessed at necropsy as previously described [19,75]. Embryos exhibiting extensive intrauterine and/or intraembryonic hemorrhage or grossly abnormal morphology were scored as nonviable. Images for gross pathologic evaluation were captured using a Canon EOS 70D Digital SLR camera or an iPhone 14 Pro Max with a 48 MP camera. Uteri were fixed in 4% neutral-buffered formalin overnight and paraffin-embedded for histological analysis. Platelet-depleted plasma was generated by centrifugation from citrated whole blood collected at euthanasia, flash-frozen in liquid nitrogen, and preserved at -80°C.

### Euthanasia and sample collection – ECM

Mice were anesthetized using 2.5% tribromoethanol (Avertin) administered via intraperitoneal injection or by isoflurane inhalation and euthanized via cervical dislocation. In some experiments, non-moribund, infection day-matched ECM- mice were euthanized for collection of blood and tissues for comparison to the ECM+ counterparts. All other non-moribund mice not exhibiting neurological symptoms were euthanized at the end of the day at 6 dpi. Brains were collected and preserved in 4% neutral-buffered formalin and paraffin-embedded for histologic assessment. Platelet-free plasma was generated by centrifugation from citrated whole blood collected at euthanasia, flash-frozen in liquid nitrogen, and preserved at -80°C.

In experiments assessing blood-brain barrier permeability, a 2% solution of Evans blue dye was prepared using Evans blue dye powder (Sigma-Aldrich, St. Louis, MO, USA) and PBS, pH 7.4 and sterile-filtered through a 0.2 μm-pore filter. At 5 dpi or upon observation of neurological signs consistent with ECM, 200μL of Evans blue dye solution per 20 g of body weight was injected intravenously and circulated for 1 hour before euthanasia. Mice were anesthetized with isoflurane and transcardially perfused with 100 mL of sterile 0.9% saline. Brains were removed and photographed using a Pixel 9 Pro. Evans blue dye was extracted from a sagittal section of brain inclusive of the cerebellum, the cerebrum and one olfactory bulb according the method of Smith et al [77], with some changes. The tissue was weighed and then minced and digested at 55^o^ C for 48 hours. Absorbance of the supernatant was measured at 620 nm with subtraction of a reading at 740 nm using a Synergy HTX plate reader, with final correction for input mass of tissue.

### Histology

Tissues were fixed in buffered formalin and transferred to ethanol prior to processing. Paraffin-embedded uteri and brains were sectioned (5μm thick) for H&E staining. Indirect immunolocalization of fibrin(ogen) was performed on unstained uterus sections using a polyclonal rabbit anti-human fibrin/fibrinogen/fibrin fragment D- and E-reactive antibody (A0080, Dako North America, Inc.), which has reactivity with mouse fibrin and fibrinogen. Micrographs were captured on a Leica Microsystems model DM4 B microscope with Leica Application Suite (LAS)-X software.

### Statistical analysis

All statistical analyses were performed using the GraphPad Prism software package (version 10.1.0). Fisher’s exact or Χ^2^ test was used for testing differences between proportions. Changes in weight, parasitemia, and hematocrit were analyzed using area under the curve (AUC) analysis. Normally distributed data for two groups were analyzed by unpaired t-test with Welch’s correction to account for unequal variances; groups of three or more were analyzed with one way ANOVA with Dunnett’s T3 post-hoc multiple comparisons test, or for two dichotomized groups, by two-way ANOVA with Fisher’s LSD test. Longitudinal data were analyzed by mixed effects ANOVA. Parasitemia data were analyzed as area under the curve (AUC). Resistance to the development of ECM was analyzed using Kaplan-Meier plots with the Mantel-Cox and Gehan-Breslow-Wilcoxon tests to assess between group differences. Differences with p ≤ 0.05 were considered significant.

## Supporting information

S1 Fig Parasitemia and hematocrit in dalteparin-treated pregnant B6 mice. (A) Percent parasitemia in PbA-infected dalteparin (Tx) or sham-treated (no Tx) B6 mice. (B) Area under the curve (AUC) of parasitemia in Tx and no Tx groups from E6.5-E12.5. (C) Percent hematocrit of Tx and no Tx groups. (D) Percent starting weight of Tx and no Tx groups at E12.5. *P < 0.05, ns = not significant, unpaired t-test with Welch’s correction (B, D).
(TIF)

S2 Fig Total embryo count in TF-intact (TF^Ctrl^) and endothelial-TF deficient (TF^Tie2Δ^) mice at E12.5. Comparisons between IP and UP groups done by two-way ANOVA, *P < 0.05, ns = not significant.
(TIF)

S3 Fig Breeding strategy evaluating TF expression on the placental trophoblast or embryo endothelium of offspring from TF^Ctrl^ crossed with TF^Tie2Δ^ mice. Transgenic male mice expressing Cre recombinase and a Tie2-specific TF deletion (TF^Tie2Δ^) are crossed with females with floxed tissue factor but no expression of Cre recombinase (TF^Ctrl^). Fetally-derived trophoblast of the resulting offspring are expected to have a genotypic distribution of ~50% TF^Ctrl^ and 50% TF^Tie2Δ^. Created in BioRender.
(TIF)

S4 Fig Alternative breeding strategy for evaluating TF expression on the placental trophoblast or embryo endothelium of offspring from TF^Tie2Δ^ dams. Transgenic male mice expressing Cre recombinase and Tie2-specific tissue factor deletion (TF^Tie2Δ^) are crossed with females of the same genotype. Fetally-derived trophoblast of the resulting offspring are expected to have a genotypic distribution of ~25% TF^Ctrl^, 25% TF^Tie2ΔΔ^ (with two Cre alleles), and 50% TF^Tie2Δ^ (with one Cre allele). Created in BioRender.
(TIF)

S1 Table ECM scoring rubric. Mouse is placed alone in a plain cage with bedding to make all observations except for limb strength, which is evaluated using a cage feed hopper; aggression, which is evaluated in a mouse restrainer; and dehydration, which requires weight measurement.
(DOCX)

S2 Table Embryo viability by dam genotype. Reported as number of viable and non-viable embryos, number of total embryos and percent viable.
(DOCX)

S1 Data Raw data associated with this manuscript. (XLSX)
