# Correction: Anticoagulant therapy and altered tissue factor expression protect against experimental placental and cerebral malaria

**DOI:** 10.1371/journal.ppat.1013624

**Published:** 2025-10-24

**Authors:** Alicer K. Andrew, Tara C. Bracken, Nicole Nazario-Maldonado, Brittany N. Russ, Kathryn T. Gonzalvo, Benjamin M. Brainard, Nigel Mackman, Julie M. Moore

The fifth author’s name is spelled incorrectly. The correct name is: Kathryn T. Gonzalvo. The correct citation is: Andrew AK, Bracken TC, Nazario-Maldonado N, Russ BN, Gonzalvo KT, Brainard BM, et al. (2025) Anticoagulant therapy and altered tissue factor expression protect against experimental placental and cerebral malaria. PLoS Pathog 21(7):e1013259. https://doi.org/10.1371/journal.ppat.1013259

There are errors in the Author Contributions. The correct contributions are

**Conceptualization:** Julie M. Moore

**Data curation** Alicer K. Andrew, Brittany N. Russ, Kathryn T. Gonzalvo

**Formal analysis** Alicer K. Andrew, Julie M. Moore

**Funding acquisition:** Julie M. Moore

**Investigation** Alicer K. Andrew, Tara C. Bracken, Nicole Nazario-Maldonado, Julie M. Moore

**Methodology** Tara C. Bracken, Nicole Nazario-Maldonado, Benjamin M. Brainard, Nigel Mackman

**Resources** Nigel Mackman

**Supervision** Julie M. Moore

**Visualization** Alicer K. Andrew, Tara C. Bracken, Julie M. Moore

**Writing – original draft** Alicer K. Andrew, Tara C. Bracken

**Writing – review & editing** Alicer K. Andrew, Tara C. Bracken, Benjamin M. Brainard, Nigel Mackman, Julie M. Moore
