# Supplementary figures and images for: Anticoagulant therapy and altered tissue factor expression protect against experimental placental and cerebral malaria
Source: PLoS Pathog. 2025 Jul 3;21(7):e1013259. doi: 10.1371/journal.ppat.1013259 (PMC12244638; doi:10.1371/journal.ppat.1013259)

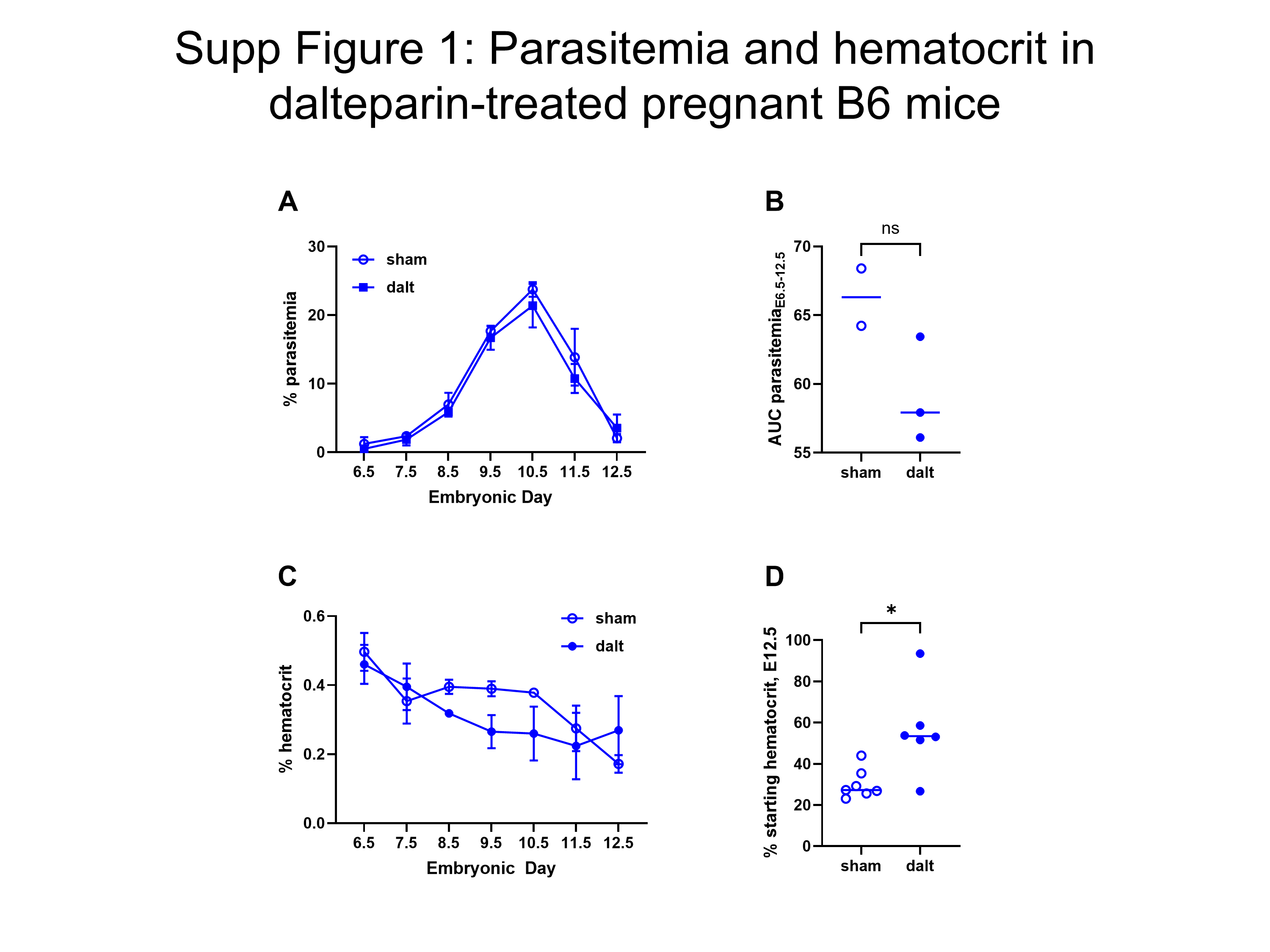

Supplement: S1 Fig — (A) Percent parasitemia in PbA-infected dalteparin (Tx) or sham-treated (no Tx) B6 mice. (B) Area under the curve (AUC) of parasitemia in Tx and no Tx groups from E6.5-E12.5. (C) Percent hematocrit of Tx and no Tx groups. (D) Percent starting weight of Tx and no Tx groups at E12.5. *P < 0.05, ns = not significant, unpaired t-test with Welch’s correction (B, D). (TIF) [file ppat.1013259.s001.tif]

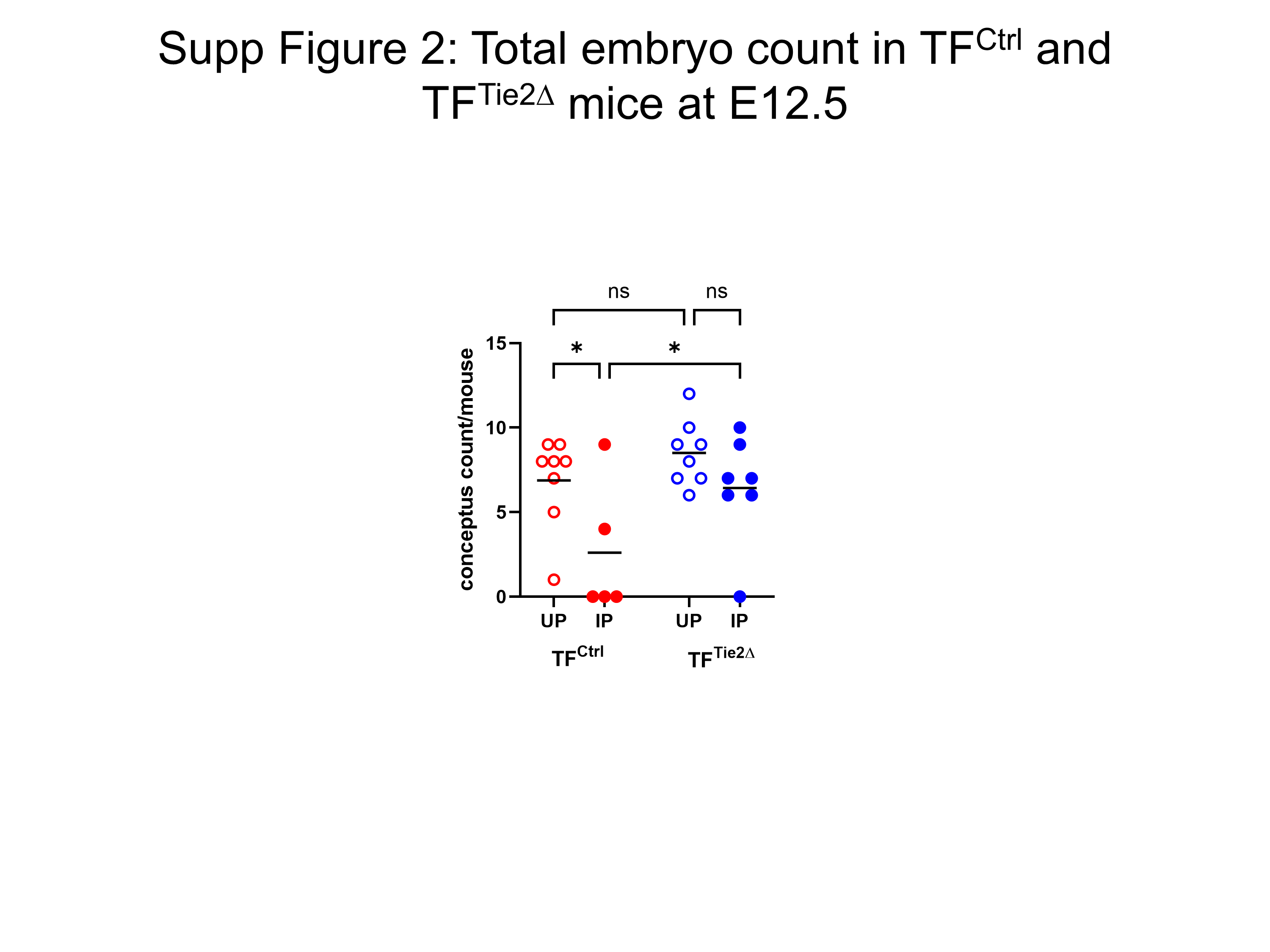

Supplement: S2 Fig — Total embryo count in TF-intact (TFCtrl) and endothelial-TF deficient (TFTie2Δ) mice at E12.5. Comparisons between IP and UP groups done by two-way ANOVA, *P < 0.05, ns = not significant. (TIF) [file ppat.1013259.s002.tif]

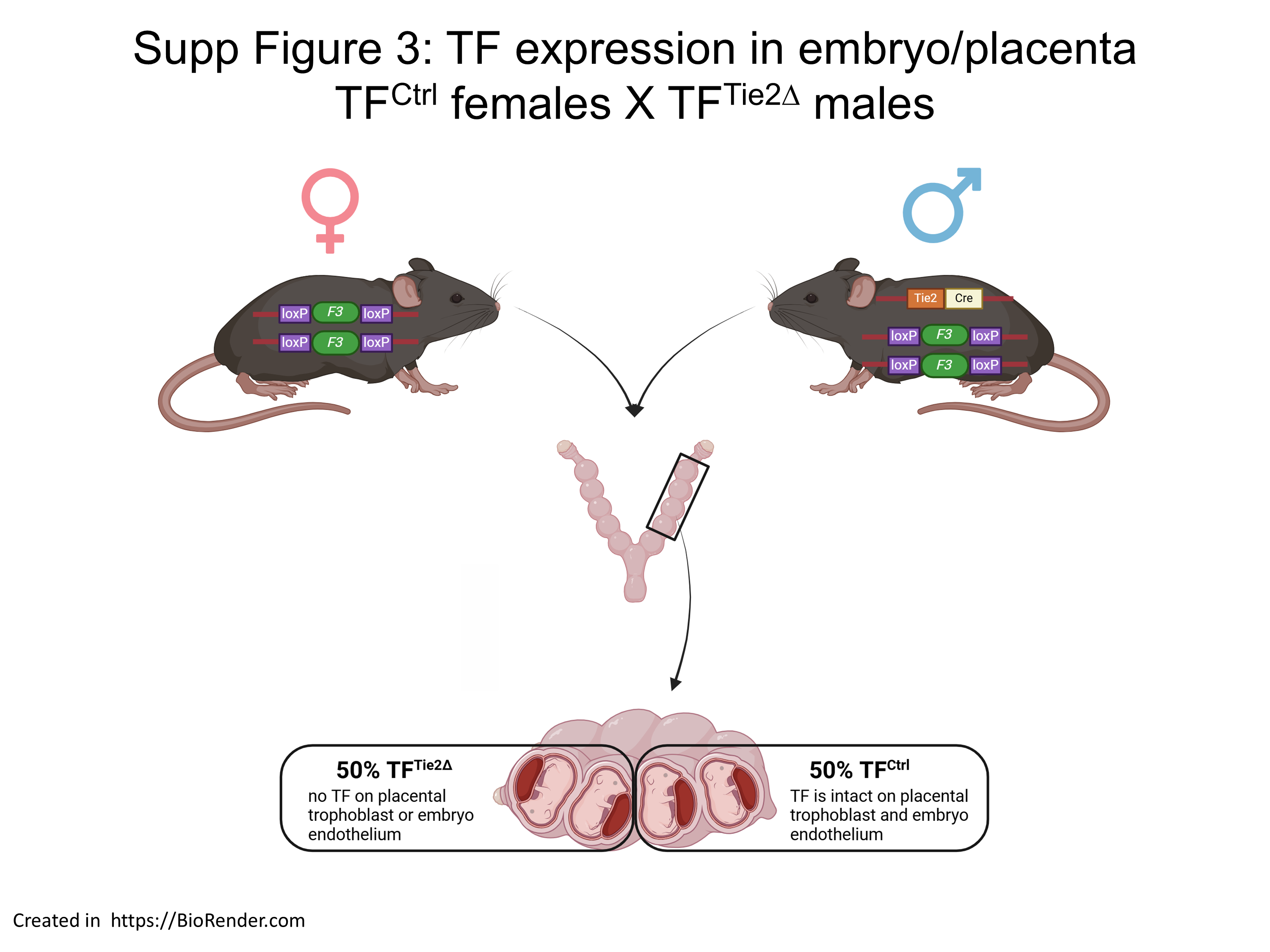

Supplement: S3 Fig — Breeding strategy evaluating TF expression on the placental trophoblast or embryo endothelium of offspring from TFCtrl crossed with TFTie2Δ mice. Transgenic male mice expressing Cre recombinase and a Tie2-specific TF deletion (TFTie2Δ) are crossed with females with floxed tissue factor but no expression of Cre recombinase (TFCtrl). Fetally-derived trophoblast of the resulting offspring are expected to have a genotypic distribution of ~50% TFCtrl and 50% TFTie2Δ. Created in BioRender. (TIF) [file ppat.1013259.s003.tif]

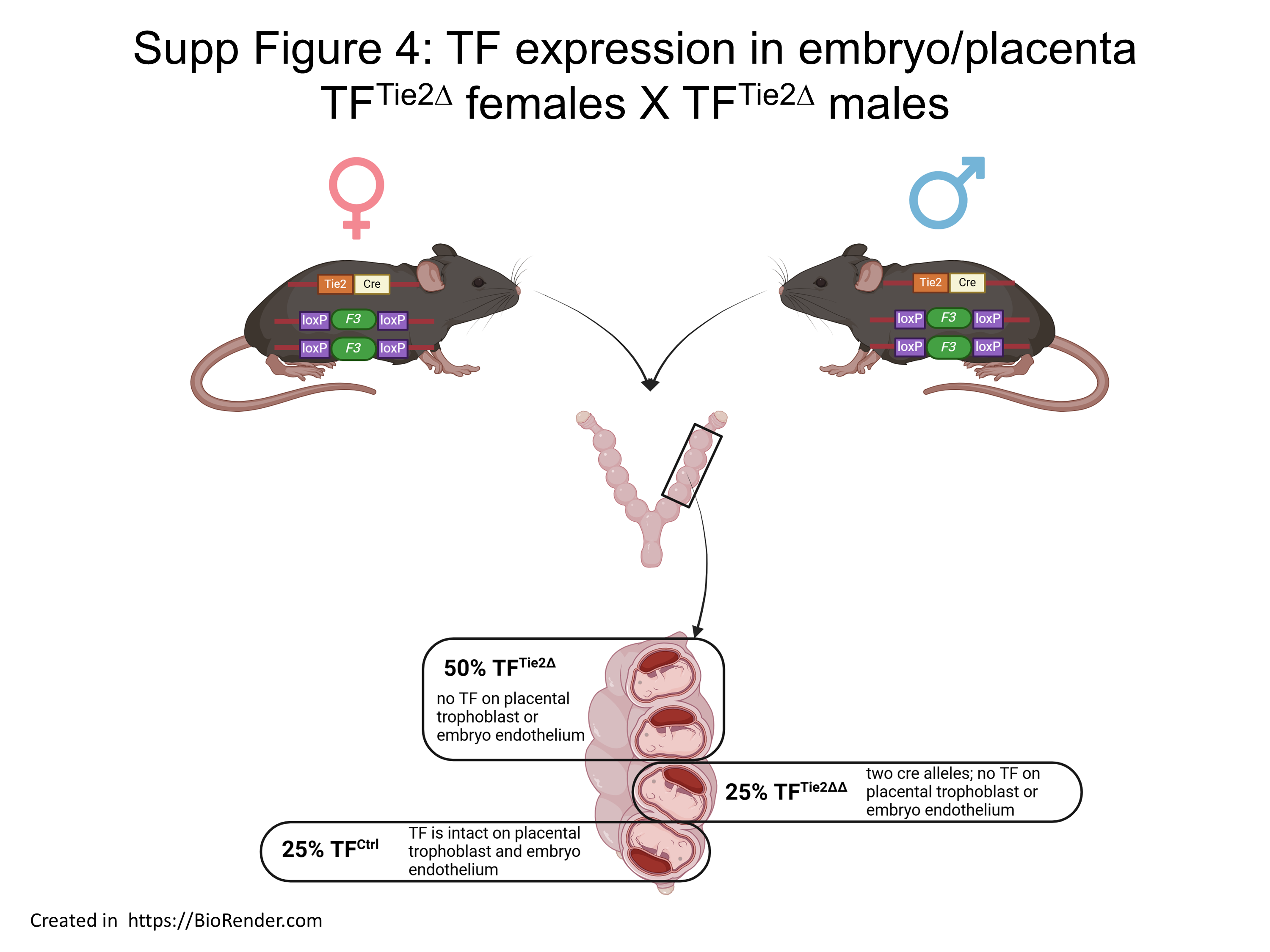

Supplement: S4 Fig — Alternative breeding strategy for evaluating TF expression on the placental trophoblast or embryo endothelium of offspring from TFTie2Δ dams. Transgenic male mice expressing Cre recombinase and Tie2-specific tissue factor deletion (TFTie2Δ) are crossed with females of the same genotype. Fetally-derived trophoblast of the resulting offspring are expected to have a genotypic distribution of ~25% TFCtrl, 25% TFTie2ΔΔ (with two Cre alleles), and 50% TFTie2Δ (with one Cre allele). Created in BioRender. (TIF) [file ppat.1013259.s004.tif]
