# Supplementary material for: Anticoagulant therapy and altered tissue factor expression protect against experimental placental and cerebral malaria
Source: PLoS Pathog. 2025 Jul 3;21(7):e1013259. doi: 10.1371/journal.ppat.1013259 (PMC12244638; doi:10.1371/journal.ppat.1013259)
Supplement: S1 Table — Mouse is placed alone in a plain cage with bedding to make all observations except for limb strength, which is evaluated using a cage feed hopper; aggression, which is evaluated in a mouse restrainer; and dehydration, which requires weight measurement. (DOCX) [file ppat.1013259.s005.docx]

S1 Table: ECM scoring rubric

Mouse is placed alone in a plain cage with bedding to make all observations except for limb strength, which is evaluated using a cage feed hopper; aggression, which is evaluated in a mouse restrainer; and dehydration, which requires weight measurement.

| **Scale** | **Limb Strength (LS)** | **Explora-tory behavior (EB)** | **Grooming (G)** | **Body Position (BP)** | **Touch escape (TE)** | **Abnormal breathing (AB)** | **Shivering (Sh)** | **Dehydra-tion** | **Aggres-sion (A)** | **Eye position (E)** |
| --- | --- | --- | --- | --- | --- | --- | --- | --- | --- | --- |
| 2 | strong pull back | explore 4 corners in 30 s | clean/sheen | full extension | instant and bilateral | normal | normal | normal | bite attempt prior to tail scab removal, in 5 seconds | open |
| 1 | weak pull back | 2-3 corners explored in 90 s | Dusty/ pilo-erection | hunched | unilateral | intermediate | intermediate | loss > 5% body weight | bite attempt with tail scab removal* | half closed |
| 0 | no grasp | none | matted, hair out of place | on side | none | severe | severe | - | none | closed |

*for blood collection to measure parasitemia and hematocrit
