# Supplementary material for: Anticoagulant therapy and altered tissue factor expression protect against experimental placental and cerebral malaria
Source: PLoS Pathog. 2025 Jul 3;21(7):e1013259. doi: 10.1371/journal.ppat.1013259 (PMC12244638; doi:10.1371/journal.ppat.1013259)
Supplement: S2 Table — Reported as number of viable and non-viable embryos, number of total embryos and percent viable. (DOCX) [file ppat.1013259.s006.docx]

S2 Table: Per mouse embryo viability

| Genotype |  | # viable embryos | # non-viable embryos | total embryos | % viable |
| --- | --- | --- | --- | --- | --- |
| TF^Ctrl^ |  | 0 | 0 | 0 | 0 |
| TF^Ctrl^ |  | 0 | 0 | 0 | 0 |
| TF^Ctrl^ |  | 0 | 0 | 0 | 0 |
| TF^Ctrl^ |  | 0 | 9 | 9 | 0 |
| TF^Ctrl^ |  | 0 | 4 | 4 | 0 |
| TF^Tie2Δ^ |  | 0 | 9 | 9 | 0 |
| TF^Tie2Δ^ |  | 0 | 0 | 0 | 0 |
| TF^Tie2Δ^ |  | 1 | 5 | 6 | 16.7 |
| TF^Tie2Δ^ |  | 6 | 1 | 7 | 85.7 |
| TF^Tie2Δ^ |  | 10 | 0 | 10 | 100 |
| TF^Tie2Δ^ |  | 6 | 0 | 6 | 100 |
| TF^Tie2Δ^ |  | 7 | 0 | 7 | 100 |
|  |  |  |  |  |  |
